# Phonon-mediated superconductivity in ${\text{Mg}}_{{1 - x}} {\text{Mo}}_{x} {\text{B}}_{2}$ compounds: a crystal prediction via cluster expansion and particle-swarm optimization

**DOI:** 10.1038/s41598-023-44632-3

**Published:** 2023-11-20

**Authors:** Prutthipong Tsuppayakorn-aek, Wei Luo, Rajeev Ahuja, Thiti Bovornratanaraks

**Affiliations:** 1 Extreme Conditions Physics Research Laboratory and Center of Excellence in Physics of Energy Materials (CE:PEM), Department of Physics, Faculty of Science, Chulalongkorn University, Bangkok, 10330 Thailand; 2 Condensed Matter Theory Group, Materials Theory Division, Department of Physics and Astronomy, Uppsala University, Box 516, SE-751 20 Uppsala, Sweden; 3 Department of Physics, Indian Institute of Technology (IIT) Ropar, Rupnagar, Punjab 140001 India

**Keywords:** Materials science, Physics

## Abstract

Investigating superconductivity represents one of the most significant phenomena in the field of condensed matter physics. Our simulations aim to elucidate the structures in the metallic state of Mg_1−x_Mo_x_B_2_, which is essential for predicting their superconducting properties. By employing a first-principle cluster expansion and particle-swarm optimization, we have predicted the structures of Mg_1−x_Mo_x_B_2_ ternary alloys, including Mg_0.667_Mo_0.333_B_2_, Mg_0.5_Mo_0.5_B_2_, and Mg_0.333_Mo_0.667_B_2_, and have determined their thermodynamically stable configurations under both atmospheric and high-pressure conditions. To investigate the potential for superconductivity in these structures, we have conducted a detailed examination of electronic properties that are pertinent to determining the superconducting state. Regarding superconducting properties, Mg_0.333_Mo_0.667_B_2_ exhibits superconductivity with a critical temperature (T_c_) of 7.4 K at ambient pressure. These findings suggest that the theoretically predicted structures in Mg/Mo-substituted metal borides could play a significant role in synthesis and offer valuable insights into superconducting materials.

## Introduction

The discovery of superconductivity in magnesium diboride with a high critical temperature (*T*_*c*_) of 39 K was a groundbreaking event^1^ , making a significant contribution to the high *T*_*c*_ superconductivity community. Following this discovery, numerous theoretical and experimental studies have been conducted in the field of superconductivity to gain a deeper understanding of the unique properties of MgB_2_. The ultimate goal is to hope that these investigations will lead to the development of new superconducting materials that can enhance technological advancements^2–6^. Significantly, the Bardeen-Cooper-Schrieffer (BCS) theory of phonon-mediated superconductivity has successfully elucidated superconducting quantum phases. This prediction presents an effective approach to understanding electron-phonon interactions, paving the way for extensive research on superconducting quantum phases. One current focus in superconductivity research is the identification and development of methods to increase the *T*_*c*_. The unique properties of boron enhance the superconducting properties of binary compounds, and it is remarkable how a multitude of research papers on BCS-based superconducting borides have emerged in recent years. This new wave of superconducting borides is driving progress in the field, as it reveals boron’s role in various systems exhibiting exotic physical and chemical properties. Examples include Ca-B^7^, Rb-B^8^, and La-B^9^ systems, where boron plays a central compositional role.

Regarding the study of superconducting properties in MgB_2_, achieving superconductivity should well be within the current capabilities of boron. According to a theoretical study by Kortus et al.^10^, the abundance of boron has been pointed out as a possible reason for MgB_2_’s superconductivity, considering both covalent B-B and ionic B-Mg bonding. It is important to note that these bonds are sufficiently strong, leading to a strong electron-phonon coupling. To the best of the researcher’s knowledge, MgB_2_ exhibits an anisotropic superconducting gap^10–15^. In more detail, Floris *el*.*al*.^14^. have shown that the Coulomb interaction affects σ and π states differently. This implies that the presence of σ and π states contributes to stabilizing the observed superconducting structure. Recent extensive studies on dense metallic borides, particularly molybdenum diboride (MoB_2_), have been conducted by Quan et al.^16^. MoB_2_ demonstrates superconductivity with a *T*_*c*_ of 32 K at a pressure of 100 GPa, possibly indicating proximity to the *T*_*c*_ of MgB_2_. As reported in the literature^16–22^, it is intriguing to further explore MgB_2_ and MoB_2_ by considering energetically stable crystal structures involving mixtures of magnesium and molybdenum. This exploration aims to uncover the properties of Mg_1−x_Mo_x_B_2_. Indeed, the impact of Mo addition to MgB_2_ has been experimentally investigated Bayazit et al.^23^. Their experimental findings suggest that the *T*_*c*_ decreases with an increase in Mo concentration. However, the actual crystal structure remains unclear, lacking atomic positions and symmetry details. Further exploration of boride compounds’ superconductivity has revealed the existence of a class of boride compounds that can superconduct at zero pressure^24^. This opens up the possibility of developing new superconducting materials based on boron. Therefore, we aim to study various Mg_1−x_Mo_x_B_2_ ternary alloys to understand the role of superconducting properties under ambient pressure. The cluster expansion and particle-swarm optimization have yielded noteworthy results, showcasing candidate structures in the metallic state of Mg_1−x_Mo_x_B_2_, including Mg_0.667_Mo_0.333_B_2_, Mg_0.5_Mo_0.5_B_2_, and Mg_0.333_Mo_0.667_B_2_. These structures contribute to our understanding of superconductivity and provide valuable insights into the study of superconducting quantum phases within such materials. This work’s results underscore the potential of cluster expansion and particle-swarm optimization as tools for identifying promising superconducting metallic alloys.

In this study, our objective is to provide essential insights into the structural behavior and electronic properties of the metallic state of Mg_1−x_Mo_x_B_2_ under varying pressures. We are on a quest to identify energetically stable compositions of Mg_1−x_Mo_x_B_2_ along with relevant atomic configurations, using a combination of the cluster expansion method and first-principles calculations. The cluster expansion (CE) method, which relies on density functional theory (DFT), serves as a robust tool for exploring novel stoichiometries within Mg_1−x_Mo_x_B_2_. Our theoretical predictions have led to the discovery of new compositions. Furthermore, we can predict the ground-state structure by assessing the formation energy of a given configuration at ambient pressure. Additionally, we have reported on the high-pressure phase of Mg_0.5_Mo_0.5_B_2_ through the particle-swarm optimization (PSO) algorithm. This simulation aims to shed light on how these alloys behave under varying pressure levels, taking into account spin-orbit coupling (SOC) calculations. Following the path to understanding superconductivity, we have calculated the electron-phonon coupling (EPC) parameter λ using the isotropic Eliashberg function and employed the Allen-Dynes modified McMillan equation to assess the value of *T*_*c*_. Lastly, we’ve discussed the potential impact of electronic properties on the calculated superconducting phases.

## Methods

In relation to the crystallography of Mg_1−x_Mo_x_B_2_, we conduct a comprehensive investigation of precise stoichiometries under atmospheric pressure employing a CE technique. The CE method, which combines various atomic configurations to create larger structures, may not consistently result in the lowest enthalpy. It is important to note that CE has limitations in discovering novel structures. Therefore, as a potential approach to achieve novel structures with lower enthalpy values, particularly for superconducting materials, we might consider employing the PSO algorithm within an evolutionary framework based on DFT. PSO is an advanced analytical technique that offers effective solutions for determining crystal structures under high pressure. Its precision and reliability have enabled us to accurately predict the structural characteristics of various systems, including previously unknown ones. Thanks to the capabilities of this method, significant discoveries in crystal structures have been made, especially in high-pressure conditions. Additionally, our investigation into the crystallography of Mg_1−x_Mo_x_B_2_ will delve deeper into their structural stability, taking into account the influence of SOC. Moreover, we will identify the electronic properties of the new superconducting quantum phases by examining their partial density of states (PDOS). Furthermore, it is worth noting that dynamic stability plays a crucial role in both creating and maintaining superconducting quantum phases in Mg_1−x_Mo_x_B_2_. To summarize this work, we provide a detailed analysis of our study of superconducting properties in the following subsections.

### Structural predictions

In the context of this study, we successfully predicted all configurations of Mg_1−x_Mo_x_B_2_ using the first-principles CE method initially proposed by Sanchez et al.^25^, at ambient pressure. The CE method was implemented through the MIT Ab initio Phase Stability (MAPS) code^26^, which is part of the Alloy-Theoretic Automated Toolkit (ATAT)^27^. Candidate structures, including stable and metastable ground-state configurations, were validated through first-principles calculations based on DFT, utilizing the Quantum Espresso (QE) package^28,29^. These calculations employed an energy cutoff of 80 Ry and 4000 k-point meshes. To initiate the generation of structural configurations, we explored 80 configurations for Mg_1−x_Mo_x_B_2_. Subsequently, we investigated the derived metastable ground-state structures in Mg_1−x_Mo_x_B_2_ using the PSO approach for structural predictions. This was implemented through the Crystal structure AnaLYsis by Particle Swarm Optimization (CALYPSO) method^30,31^ the Vienna ab initio simulation package (VASP) code^32^. In our search for favorable structures of Mg_1−x_Mo_x_B_2_, we considered cell sizes of up to 4 formula units (f.u.).

### Structural stability and electronic structure

For optimized calculations, the electronic band structure and density of states are computed using first-principles calculations based on DFT as implemented in the VASP^32^. We employed the generalized gradient approximation (GGA), specifically the Perdew, Burke, and Ernzerhof (PBE) exchange-correlation functional^33^, along with the conjugate gradient scheme for these computations. Details of the calculations for all structures neglected entropy contributions since they were performed at 0 K, where the formation energy suffices to confirm phase stability. The projector augmented wave (PAW) method^34^ was used with valence electrons of 2*s* 2*p* 3*s*, 4*s* 4*p* 4*d* 5*s*, and 2*s* 2*p*, for Mg, Mo, and B atoms, respectively. The pseudocore radii for Mg, Mo, and B atoms were 1.7, Bohr 2.5 Bohr, and 1.1 Bohr, respectively, ensuring no sphere overlap under compression. Optimizing the structures involved a plane-wave basis set with a cutoff energy of 500 eV and an initial Brillouin Zone (BZ) sampling grid spacing of 2π ×0.02 Å^−1^. To account for the effect of SOC with scalar-relativistic eigenfunctions, as implemented in the VASP code^32^, SOC was included in all simulations of the various phases of Mg$_{1-x}$Mo$_{x}$B$_{2}$^35,36^. This is particularly relevant since Mo, being one of the heavy elements, plays a significant role in stabilizing Mg_1−x_Mo_x_B_2_ across the range of 0 < x < 1 for Mo concentration^37^.

### Dynamic stability and phonon-mediated superconductivity

Phonon calculations were conducted using first-principles lattice dynamics and density-functional perturbation theory (DFPT) as implemented in the PHONOPY package^38,39^, in conjunction with the VASP code. For critical temperature superconductivity calculations, EPC and spectral function computations were also performed using DFPT^28^. A plane-wave energy cutoff of 80 Ry was utilized, and all calculations were carried out within the GGA-PBE scheme. In the context of EPC matrix element calculations for P6/mmm Mg_0.667_Mo_0.333_B_2_, R3 ¯m Mg_0.333_Mo_0.667_B_2_, Immm Mg_0.5_Mo_0.5_B_2_, and I4 ¯m2 Mg_0.5_Mo_0.5_B_2_, computations were performed within the first Brillouin zone (BZ) using q-meshes of 2×2×2, 2×2×4, 2×2×2, and 2×2×1, respectively. The individual EPC matrices were utilized with k-points meshes of 16×16×24, 12×12×12, 24×24×24, and 12×12×1. It is worth noting that the Eliashberg spectral function depended on a dense k-points mesh, encompassing all k and k+q grid points, which covered the q-points mesh, as implemented in the Quantum Espresso code^29^. These conditions for q-points and the calculated spectral function align with findings from previous theoretical studies^40,41^. The Allen-Dynes (AD) equation^42^ was employed with an effective Coulomb pseudopotential parameter, $\mu ^{*}$= 0.10.

## Results and discussion

**Figure 1:**
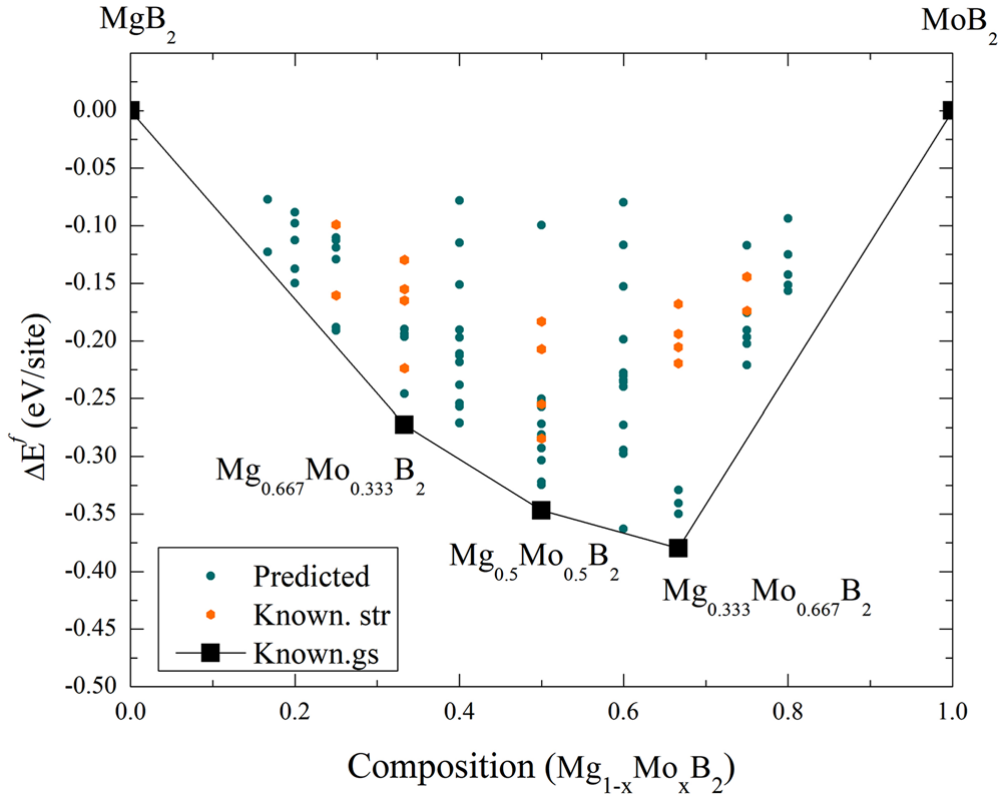
Formation energy at ambient pressure for the Mg$_{1-x}$Mo$_{x}$B$_{2}$ system, covering a range of 0 $\le$ x $\le$ 1. The term “predicted” denotes structures for which energy calculations have not been conducted via DFT, “known str” signifies structures for which DFT-based energy calculations have been performed, and “known gs” indicates ground state energies that have been validated through DFT calculations.

**Figure 2:**
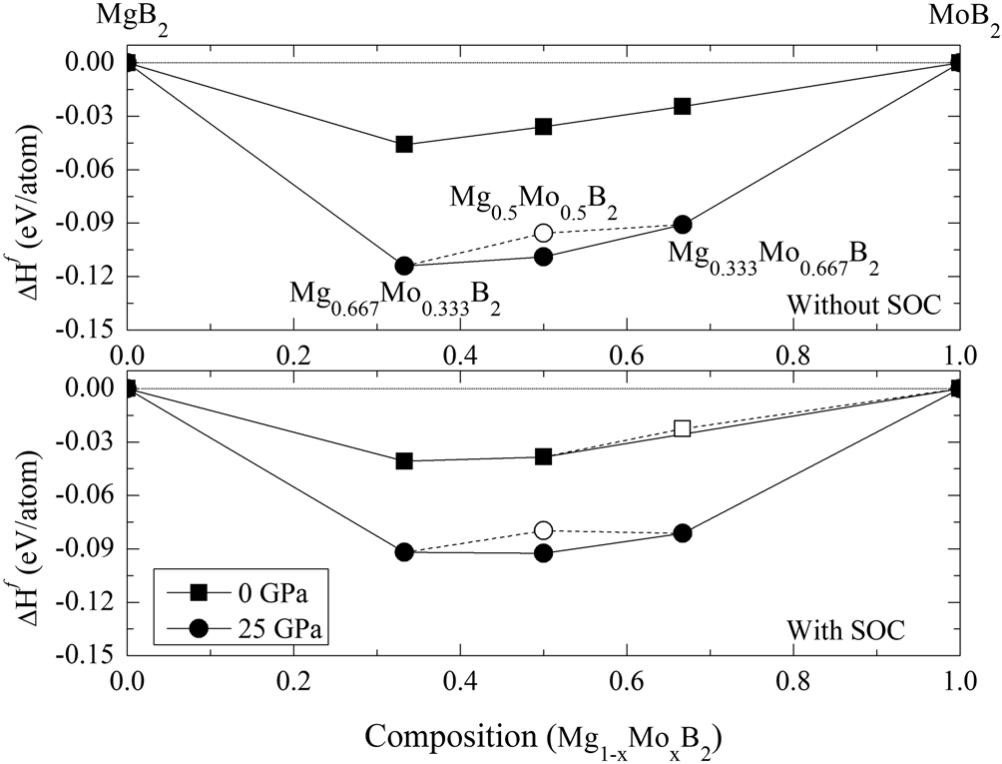
Formation enthalpy at 0 K for the Mg$_{1-x}$Mo$_{x}$B$_{2}$ system, ranging from 0 $\le$ x $\le$ 1. Circles and squares represent calculations at pressures of 0 GPa and 25 GPa. Filled circles and squares indicate stable structures, while open circles and squares represent metastable ones. Panels (a) and (b) correspond to calculations without and with spin-orbit coupling (SOC), respectively.

To explore various stoichiometries involving Mg/Mo-substituted metal borides, we employed the CE method. Thermodynamic stability of the novel Mg_1−x_Mo_x_B_2_ structures is presented under ambient conditions, where 0 $\le$ x $\le$ 1. The formation energy at ambient pressure is depicted by a convex hull in Fig. 1. Predicted structures refer to those for which energies have not yet been calculated using DFT, while known structures have their energies confirmed by DFT. Furthermore, known ground states refers to the ground state energies that have been verified through DFT. To select configurations accurately, we considered a parity plot consisting of 80 chosen configurations, which achieved a satisfactory level of accuracy with a cross- validation (CV) score^26^ amounting to 0.12 eV/site. In essence, the CE method’s solution enabled us to explore novel compositions within Mg_1−x_Mo_x_B_2_. Our primary structural prediction results unveiled ground-state structures. The current structural predictions rely on these ground-state structures, determined through the calculation of the formation energy, which is obtained as

1 $$\begin{aligned} \Delta E^{f} = E[Mg_{1-x}Mo_{x}B_{2}]-(1-x)E[MgB_{2}]-(x)E[MoB_{2}], \end{aligned}$$

in this equation, $\Delta E^{f}$ represents the formation energy, *E*[Mg$_{1-x}$Mo$_{x}$B$_{2}$] denotes the total energy of Mg_1-x_Mo_x_B_2_ with concentration in the range 0 < x < 1, *E*[MgB$_{2}$] is the total energy of MgB_2_ with a hexagonal structure, *E*[MoB$_{2}$] represents the total energy of MoB_2_ with a rhombohedral β -MoB_2_ structure. It is important to note that MgB$_{2}$ and MoB$_{2}$ in Eq. (1) correspond to the hexagonal structure and the rhombohedral $\beta$-MoB$_{2}$ structure, respectively. The results indicate that Mg_0.667_Mo_0.333_B_2_, Mg_0.5_Mo_0.5_B_2_, and Mg_0.333_Mo_0.667_B_2_ are energetically stable. This clearly confirms their stability against decomposition into MgB_2_ and MoB_2_ at ambient pressure. However, it should be mentioned that MoB_2_, initially adopted from the MgB_2_-type with a hexagonal structure (symmetry: P6/mmm), might not be the ground state structure. As a result, corresponding formation energies are normalized by the rhombohedral β -MoB_2_ structure, as shown in the results presented in Fig. 2.

According to Fig. 2, the relatively most stable structures along the convex hull are Mg$_{0.667}$Mo$_{0.333}$B$_{2}$, and Mg$_{0.5}$Mo$_{0.5}$B$_{2}$, and Mg$_{0.333}$Mo$_{0.667}$B$_{2}$. These candidate structures underwent structural relaxation calculations based on DFT without SOC at zero pressure. However, the present results indicate that Mg$_{0.333}$Mo$_{0.667}$B$_{2}$ experiences slight destabilization at ambient pressure when SOC is considered. The overall impact of SOC on x = 0.667 is not very pronounced. When Mg$_{0.667}$Mo$_{0.333}$B$_{2}$ and Mg$_{0.333}$Mo$_{0.667}$B$_{2}$ are subjected to computational compression, both structures remain thermodynamically stable at a pressure of 25 GPa, as confirmed by calculations performed both with and without SOC. However, notable differences emerge in the case of Mg$_{0.5}$Mo$_{0.5}$B$_{2}$. It suggests that Mg$_{0.5}$Mo$_{0.5}$B$_{2}$ is thermodynamically metastable at the pressure of 25 GPa, as indicated by its position above the convex hull between Mg$_{0.667}$Mo$_{0.333}$B$_{2}$ and Mg$_{0.333}$Mo$_{0.667}$B$_{2}$ in Fig. 2. At this stage, one possible approach to achieve a stable structure in Mg$_{0.5}$Mo$_{0.5}$B$_{2}$ is to consider predicting its crystallography using the PSO algorithm within an evolutionary framework based on DFT. The PSO method is a powerful tool for high-pressure systems and has effectively predicted stable ground-state structures. Consequently, we can compare the results from both methods (CE and PSO) using formation enthalpy as a basis.

**Figure 3:**
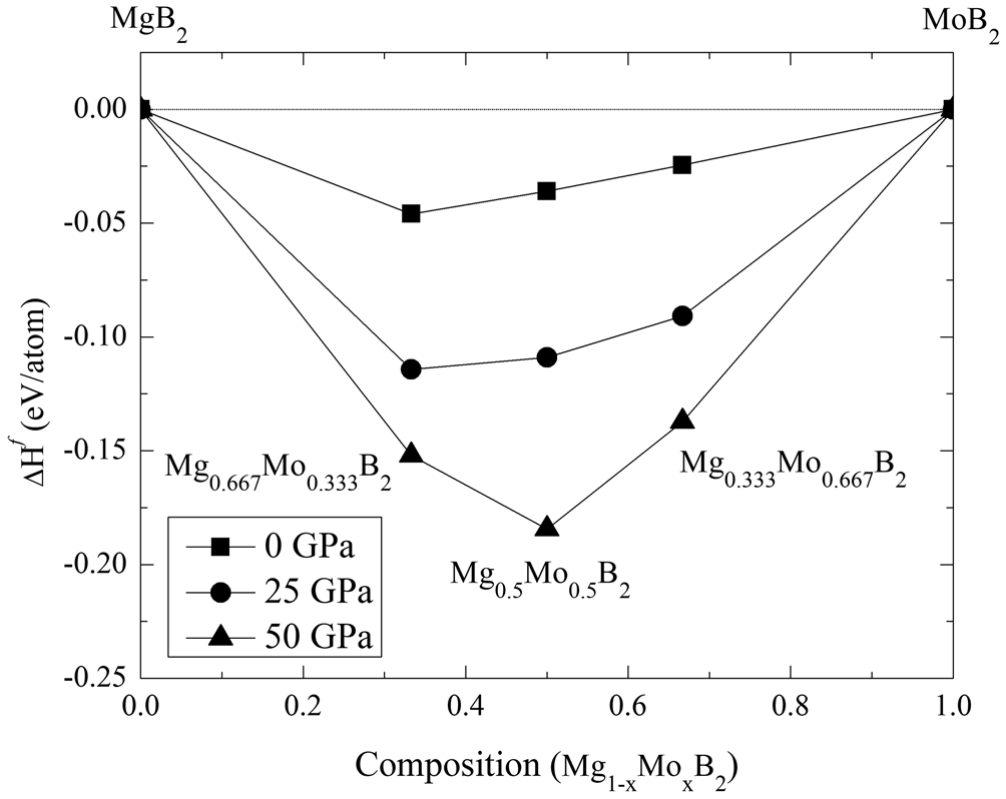
Formation enthalpy at temperature of 0 K of Mg$_{1-x}$Mo$_{x}$B$_{2}$ system, spanning from 0 ≤ x ≤ 1. Circles, squares, and triangles denote calculations at pressures of 0 GPa, 25 GPa, and 50 GPa, respectively. Filled circles and squares represent stable structures.

**Figure 4:**
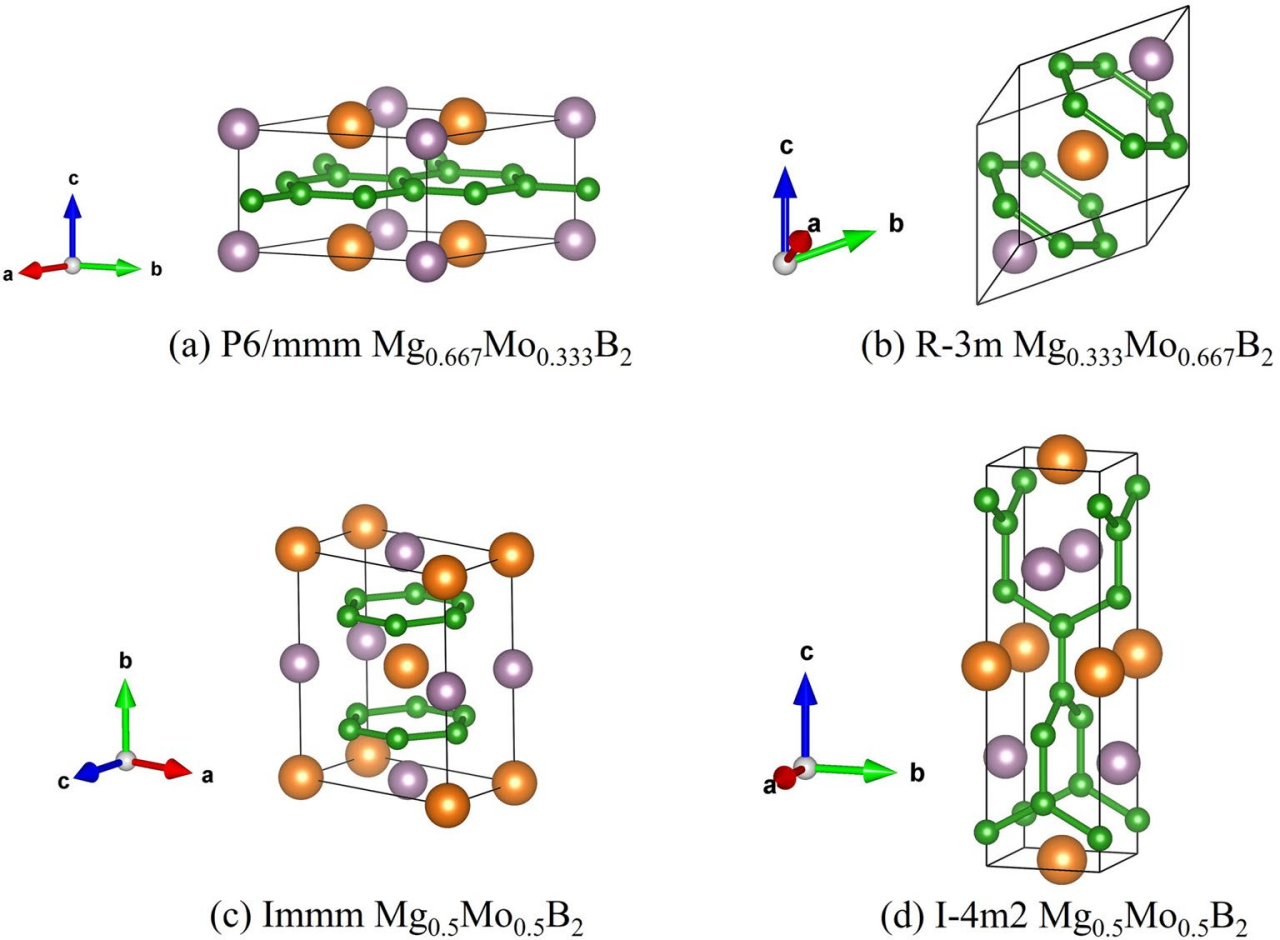
The ground-state structures of the *P*6/*mmm* Mg$_{0.667}$Mo$_{0.333}$B$_{2}$, the $R{\bar{3}}m$ Mg$_{0.333}$Mo$_{0.6667}$B$_{2}$, the *Immm* Mg$_{0.5}$Mo$_{0.5}$B$_{2}$, and the $I{\bar{4}}m2$ Mg$_{0.5}$Mo$_{0.5}$B$_{2}$; where orange, purple, and green spheres, respectively, represent Mg, Mo, and B atoms.

As discussed previously regarding the calculations involving SOC, our results indicate that the influence of SOC on the structures of $Mg_{1-x}Mo_xB_2$ ternary alloys, including Mg$_{0.667}$Mo$_{0.333}$B$_{2}$, Mg$_{0.5}$Mo$_{0.5}$B$_{2}$, and Mg$_{0.333}$Mo$_{0.667}$, especially at high pressure, is not significant. Therefore, for all considered structures, our simulations are conducted without including SOC. Now, let’s focus on the stable structure of Mg$_{0.5}$Mo$_{0.5}$B$_{2}$ at the pressure of 25 GPa, which is predicted to have a tetragonal structure with the space group $I{\bar{4}}m2$. With this result, we constructed the convex hull, displaying the formation enthalpy. Herein, we observed that the $I{\bar{4}}m2$ structure of Mg$_{0.5}$Mo$_{0.5}$B$_{2}$ is thermodynamically more favorable than the *Immm* structure of Mg$_{0.5}$Mo$_{0.5}$B$_{2}$ by approximately -0.013 eV/atom. The open circle represents the CE calculation, while the solid circle represents the PSO calculation, as shown in Fig. 2. Furthermore, we found that cluster expansion is generally a reliable method for determining stoichiometry; however, in this particular case, it may not have yielded the lowest enthalpy^43^. Utilizing the PSO technique, a thorough exploration of atomic configurations aims to pinpoint the one with the lowest enthalpy, symbolizing heightened stability and favorable thermodynamics. Consequently, we accentuate the significance of the $I{\bar{4}}m2$ Mg$_{0.5}$Mo$_{0.5}$B$_{2}$ structure, shedding light on its potential applications. This particular structure, with its distinction as the lowest-enthalpy configuration, guarantees stability even under conditions of compression. The utmost significance of its lowest enthalpy becomes evident when considering the requirement for resilient structures that can endure compression. Considering the paramount importance of stability in the $I{\bar{4}}m2$ structure of Mg$_{0.5}$Mo$_{0.5}$B$_{2}$, it becomes particularly valuable as it can maintain its form even when subjected to high levels of compression. Demonstrably, the $I{\bar{4}}m2$ Mg$_{0.5}$Mo$_{0.5}$B$_{2}$ structure resists decomposition into MgB$_{2}$ and MoB$_{2}$, even under extreme pressures beyond 25 GPa up to 50 GPa, as depicted in Fig. 3. These findings are substantiated by thermodynamic calculations, with further discussions on dynamical stability to be addressed in subsequent phonon calculations. Figure 4 visually represents  the P6/mmm Mg$_{0.667}$Mo$_{0.333}$B$_{2}$, the $R{\bar{3}}m$ Mg$_{0.333}$Mo$_{0.6667}$B$_{2}$, the *Immm* Mg$_{0.5}$Mo$_{0.5}$B$_{2}$, and the $I{\bar{4}}m2$ Mg$_{0.5}$Mo$_{0.5}$B$_{2}$. Detailed structural parameters for these configurations are provided in Table 1.

**Table 1:** The optimized structural parameters of Mg$_{1-x}$Mo$_{x}$B$_{2}$ obtained from the first-principles calculations. .

Space group	Pressure (GPa)	Lattice parameters (Å, $^{\circ }$)	Atomic coordinates (fractional)	Site
*P*6/*mmm*	0	a = 5.278 b = 5.278 c = 3.296 $\alpha$ = 90 $\beta$ = 90 $\gamma$ = 120	Mg (0.333, 0.667, 1.000)	2c
			Mo (1.000, 1.000, 1.000)	1a
			B (1.000, 0.329 0.500)	6k
$R{\bar{3}}m$	0	a = 4.501 b = 4.501 c = 4.501 $\alpha$ = 70.59 $\beta$ = 70.59 $\gamma$ = 70.59	Mg (0.500, 0.500, 0.500)	1b
			Mo (1.178, 1.178, 1.178)	2c
			B (0.329, 0.670, 1.000)	6f
*Immm*	0	a = 5.253 b = 6.595 c = 3.016 $\alpha$ = 90 $\beta$ = 90 $\gamma$ = 90	Mg (0.500, -0.500, -0.500)	2a
			Mo (0.500, 0.000,-0.500)	2d
			B (-0.162, -0.248, -0.500)	8n
$I{\bar{4}}m2$	25	a = 3.038 b = 3.038 c = 10.310 $\alpha$ = 90 $\beta$ = 90 $\gamma$ = 90	Mg (0.000, 0.000, 0.500)	2b
			Mo (0.000, 0.500, 0.750)	2d
			B (0.000, 0.000, 0.915)	4e
			B (0.500, 0.000, 0.664)	4f

**Figure 5:**
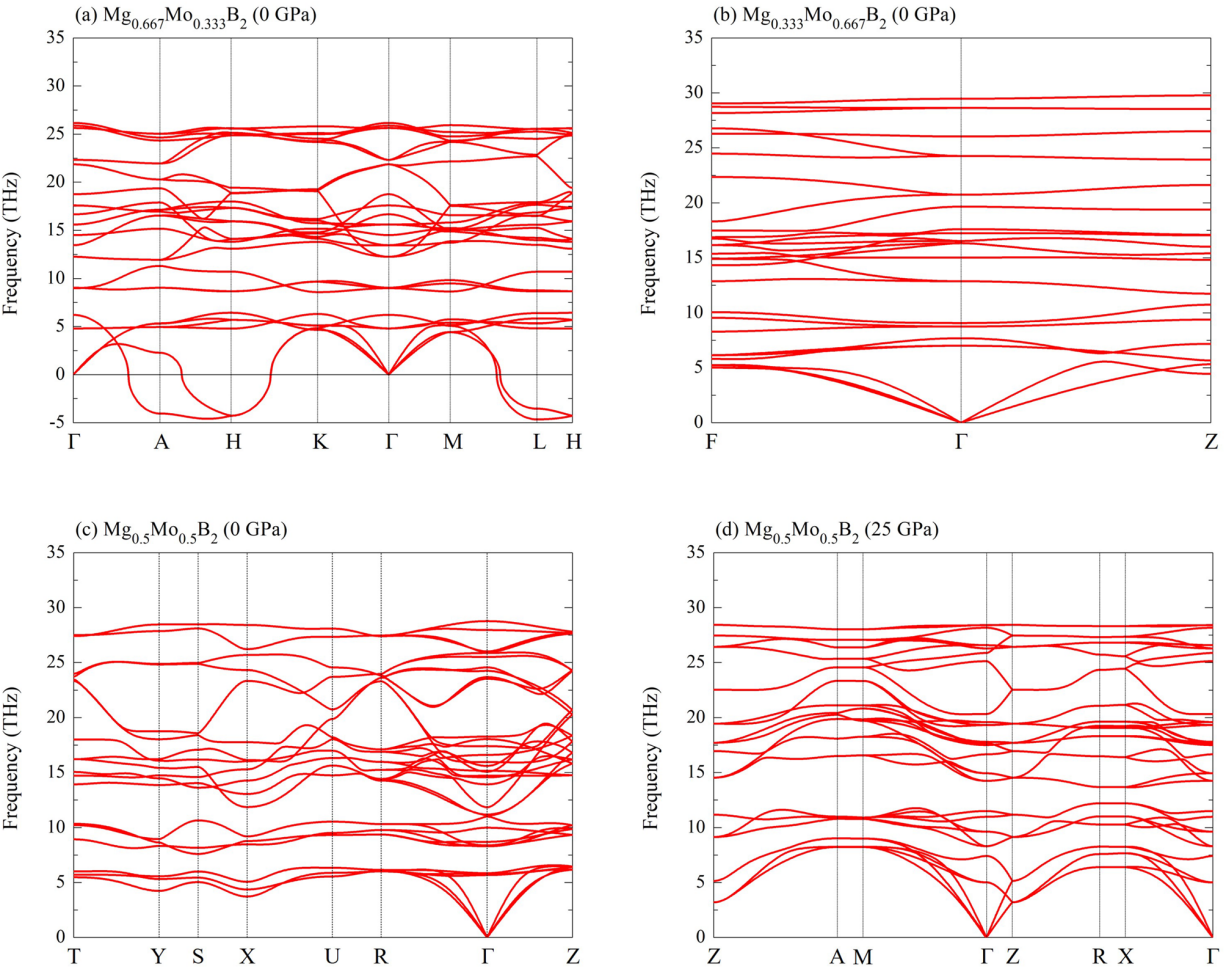
Phonon dispersion relations for: (**a**) the *P*6/*mmm* Mg$_{0.667}$Mo$_{0.333}$B$_{2}$ at ambient pressure, (**b**) the $R{\bar{3}}m$ Mg$_{0.333}$Mo$_{0.667}$B$_{2}$ at ambient pressure, (**c**) the *Immm* Mg$_{0.5}$Mo$_{0.5}$B$_{2}$ at ambient pressure, and (**d**) the $I{\bar{4}}m2$ Mg$_{0.5}$Mo$_{0.5}$B$_{2}$ at the pressure of 25 GPa.

At this juncture, it is noteworthy to reiterate that, under ambient pressure conditions, the $R{\bar{3}}m$ Mg$_{0.333}$Mo$_{0.667}$B$_{2}$, *P*6/*mmm* Mg$_{0.667}$Mo$_{0.333}$B$_{2}$, and *Immm* Mg$_{0.5}$Mo$_{0.5}$B$_{2}$ structures all exhibit negative formation relative to MgB$_{2}$ and MoB$_{2}$. However, it’s crucial to emphasize that this alone does not guarantee structural stability. To address this concern, we have conducted an investigation into their dynamical stability. Consequently, we found that the *P*6/*mmm* Mg$_{0.667}$Mo$_{0.333}$B$_{2}$ structure is dynamically unstable due to the presence of imaginary frequencies around high symmetry points (A, H, and L), as illustrated in Fig  5(a). This implies that the *P*6/*mmm* Mg$_{0.667}$Mo$_{0.333}$B$_{2}$ configuration is indeed a metastable structure. However, it is important to acknowledge that the possibility of synthesis remains, as approximately 20$\%$ of materials have been successfully obtained from metastable structures^44,45^. Furthermore, it is worth noting that the results presented here are based on calculations within the harmonic approximation. Given that this structure is already stable in the thermodynamic sense, it suggests the potential for achieving an anharmonic state^46^. Additionally, we emphasize the need for further investigation into the *P*6/*mmm* Mg$_{0.667}$Mo$_{0.333}$B$_{2}$ structure by considering the effects of thermally excited lattice dynamics on its stability. For example, the theoretical explanation of a simple cubic structure in calcium^47–49^ is well corroborated by experimental observation^50^. We have identified two dynamically stable structures, namely, the R${\bar{3}}$m Mg$_{0.333}$Mo$_{0.667}$B$_{2}$ and the *Immm* Mg$_{0.5}$Mo$_{0.5}$B$_{2}$, as presented in Figs. 5(b) and (c). Furthermore, the previously mentioned theoretical findings, which are based on formation enthalpy, reveal that the $I{\bar{4}}m2$ Mg$_{0.5}$Mo$_{0.5}$B$_{2}$ structure becomes stabilized under compression. Additionally, we have demonstrated the dynamic stability of the $I{\bar{4}}m2$ Mg$_{0.5}$Mo$_{0.5}$B$_{2}$ at a pressure of 25 GPa, as illustrated in Fig. 5(d), since it does not exhibit any imaginary frequencies. These findings suggest an intriguing avenue for exploring novel superconductivity from the perspective of a dynamically stable structure.

**Figure 6:**
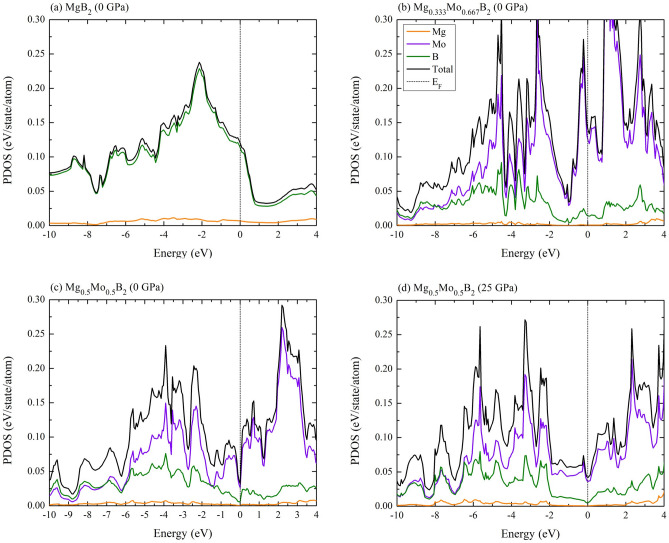
Density of states under different pressures: (**a**) the P6/mmm MgB_2_ at ambient pressure, (b) the $R{\bar{3}}m$ Mg$_{0.333}$Mo$_{0.667}$B$_{2}$ at ambient pressure, (**c**) the *Immm* Mg$_{0.5}$Mo$_{0.5}$B$_{2}$ at ambient pressure, and (**d**) the $I{\bar{4}}m2$ Mg$_{0.5}$Mo$_{0.5}$B$_{2}$ at the pressure of 25 GPa.

To unveil the exceptional properties of the Mg$_{1-x}$Mo$_{x}$B$_{2}$, it is imperative to assess the density of states (DOS) for $R{\bar{3}}m$ Mg$_{0.333}$Mo$_{0.667}$B$_{2}$, the *Immm* Mg$_{0.5}$Mo$_{0.5}$B$_{2}$, and the $I{\bar{4}}m2$ Mg$_{0.5}$Mo$_{0.5}$B$_{2}$. This assessment involves the estimation of the PDOS for these stable structures in comparison to the P6/mmm MgB_2_, as depicted in Figs. 6(a), 6(b), 6(c), and 6(d), respectively. As a consequence, we have determined that all these borides exhibit metallic properties due to the presence of electronic states at the Fermi level. Furthermore, it is noteworthy that the theoretical results indicate a slightly lower contribution of boron atoms to the electronic states in the $R{\bar{3}}m$ Mg$_{0.333}$Mo$_{0.667}$B$_{2}$, the *Immm* Mg$_{0.5}$Mo$_{0.5}$B$_{2}$, and the $I{\bar{4}}m2$ Mg$_{0.5}$Mo$_{0.5}$B$_{2}$ structures compared to the P6/mmm MgB$_{2}$. Let’s delve deeper into the electronic characteristics of the $R{\bar{3}}m$ Mg$_{0.333}$Mo$_{0.667}$B$_{2}$, the *Immm* Mg$_{0.5}$Mo$_{0.5}$B$_{2}$, and the $I{\bar{4}}m2$ Mg$_{0.5}$Mo$_{0.5}$B$_{2}$ structures. Notably, significant differences in the contributions of Mo and B atoms to the electronic structure are observed at the Fermi level. Therefore, understanding the role of Mo atoms in their structural stability is crucial when prioritizing research into superconductivity. Furthermore, it is noteworthy that the significant DOS around the Fermi level in the R${\bar{3}}$m Mg$_{0.333}$Mo$_{0.667}$B$_{2}$ is primarily attributed to Mo atoms. In contrast, the *Immm* Mg$_{0.5}$Mo$_{0.5}$B$_{2}$ and the $I{\bar{4}}m2$ Mg$_{0.5}$Mo$_{0.5}$B$_{2}$ structures exhibit a smaller DOS originating from Mo atoms. These characteristics of Mo atoms may indicate their potential as superconductors. In our exploration of superconducting properties, we conducted a manual examination of the Eliashberg spectral function $\alpha ^{2} F$($\omega$)^51^:

2 $$\begin{aligned} \alpha ^{2}F(\omega ) = \frac{1}{N_(E_F)} \sum _{\nu ,k,k'}|g^{\nu }_{kk'}|^2\delta (\epsilon _k)\delta (\varepsilon _k')\delta (\omega -\omega _{q\nu }), \end{aligned}$$

where $N_{F}$, $\epsilon _{k}$, and g$^{\nu }_{kk'}$ denote the DOS at $E_F$, the e-ph matrix element between two electronic states of wave vector k and k$^{'}$, the energy eigenvalue of the Kohn-Sham state with respect to the Fermi level, the electron-phonon matrix element for the scattering between the electronic states *k* and $k'$ via a phonon with wave vector of $q = k'-k$, while $\omega _{q\nu }$ indicate the phonon frequencies. Herein, it ought to be noted that the DOS is associated with Eliashberg spectral function in Eq.  2. The substantial total DOS at the Fermi level observed here can be primarily attributed to the magnitude of the electron-phonon matrix element. A comprehensive theoretical explanation of this phenomenon can be found in Ref^52^, shedding light on why the EPC varies in magnitude among these borides, thereby influencing the enhancement of their *T*_*c*_. As a result, the estimated DOS values at the Fermi level for $R{\bar{3}}m$ Mg$_{0.333}$Mo$_{0.667}$B$_{2}$, *Immm* Mg$_{0.5}$Mo$_{0.5}$B$_{2}$ and $I{\bar{4}}m2$ Mg$_{0.5}$Mo$_{0.5}$B$_{2}$ is estimated to be 0.138, 0.03, and 0.04 eV/state/atom, respectively. To elucidate the influence of the DOS at the Fermi level on the calculated superconducting properties and *T*_*c*_ enhancement, we will delve into this aspect in detail below.

**Table 2:** Electron-phonon interaction and logarithmic averages of phonon frequencies. The T$_{c}$s are calculated using the isotropic Eliashberg equations. A $\mu ^{*}$ = 0.10 is used.

Phase	Pressure (GPa)	$\lambda$	$\omega _{log}$	$T_c$ (K)
MgB$_{2}$ (P6/mmm)$^{1}$	0	0.80	737	34
MgB$_{2}$ (P6/mmm)$^{2}$				39
Mg$_{0.333}$Mo$_{0.667}$B$_{2}$ (R${\bar{3}}$m)$^{1}$	0	0.62	298	7.4
Mg$_{0.333}$Mo$_{0.667}$B$_{2}$ (R${\bar{3}}$m)$^{1}$	25	0.52	373	5.3
Mg$_{0.333}$Mo$_{0.667}$B$_{2}$ (R${\bar{3}}$m)$^{1}$	50	0.50	415	5.2
Mg$_{0.5}$Mo$_{0.5}$B$_{2}$ (Immm)$^{1}$	0	0.32	460	0.43
Mg$_{0.5}$Mo$_{0.5}$B$_{2}$ (I${\bar{4}}$m2)$^{1}$	25	0.36	506	1.12
Mg$_{0.5}$Mo$_{0.5}$B$_{2}$ (I${\bar{4}}$m2)$^{1}$	50	0.32	575	0.53

$$^{1}$$This work.

$$^{2}$$Reference^1^.

**Figure 7:**
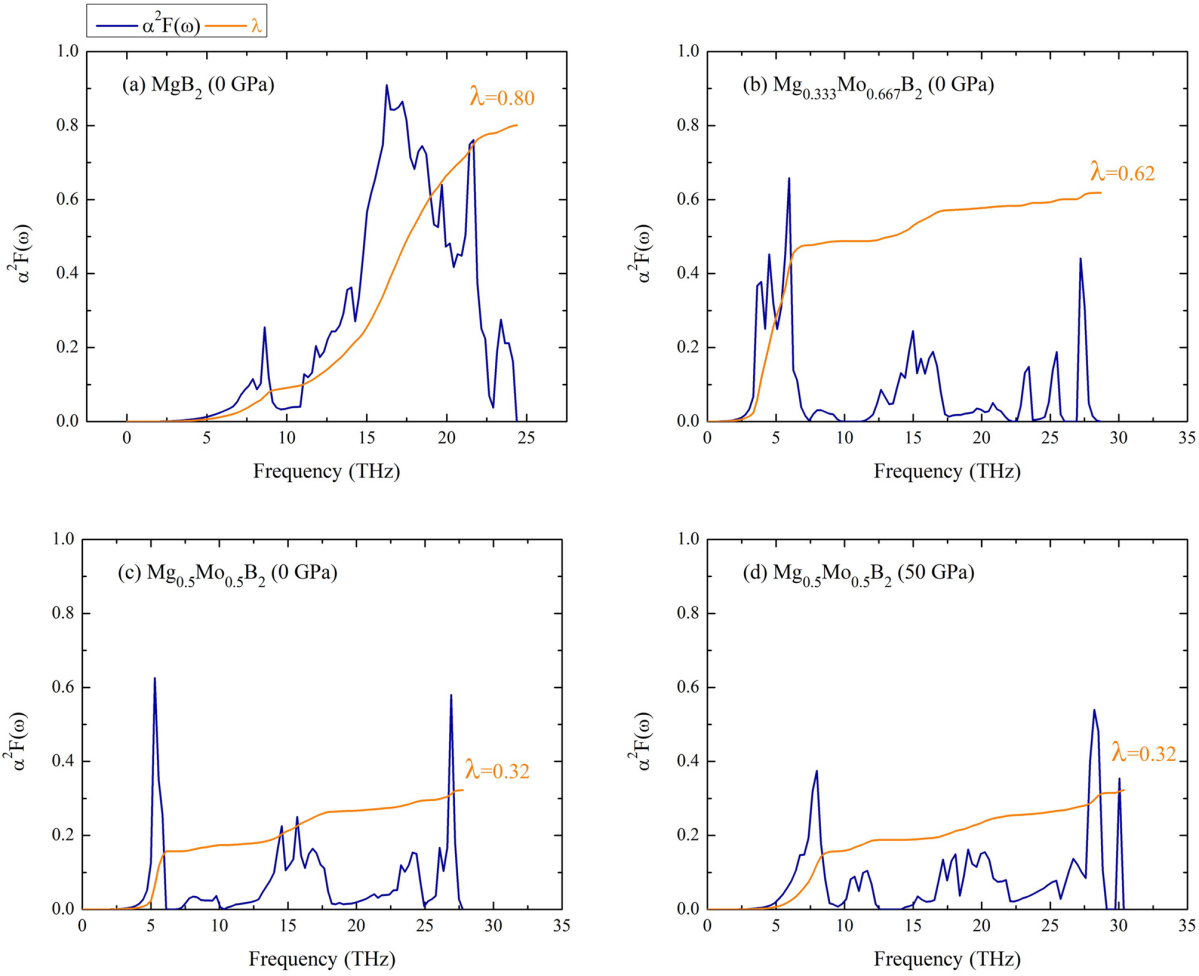
The spectral function as a function of frequency: (**a**) the P6/mmm MgB_2_ at ambient pressure, (b) the $R{\bar{3}}m$ Mg$_{0.333}$Mo$_{0.667}$B$_{2}$ at ambient pressure, (**c**) the *Immm* Mg$_{0.5}$Mo$_{0.5}$B$_{2}$ at ambient pressure, and (**d**) the $I{\bar{4}}m2$ Mg$_{0.5}$Mo$_{0.5}$B$_{2}$ at the pressure of 25 GPa.

**Figure 8:**
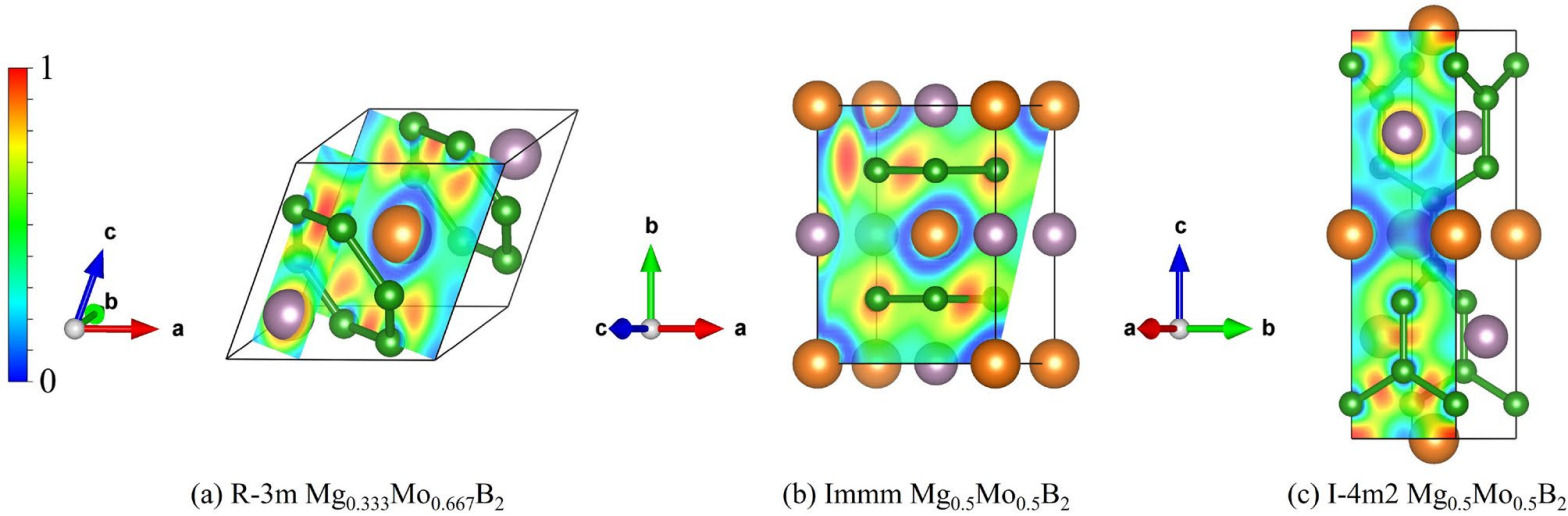
The electron localization function: (**a**) $R{\bar{3}}m$ Mg$_{0.333}$Mo$_{0.667}$B$_{2}$ at ambient pressure, (**b**) the *Immm* Mg$_{0.5}$Mo$_{0.5}$B$_{2}$ at ambient pressure, and (**c**) the $I{\bar{4}}m2$ Mg$_{0.5}$Mo$_{0.5}$B$_{2}$ at the pressure of 50 GPa.

Regarding the Eliashberg spectral function ($\alpha ^{2} F$($\omega$)) as depicted in Fig. 7, we initially examine the outcome for the P6/mmm MgB$_{2}$ at ambient condition. The $\alpha ^{2} F$($\omega$) profile of the P6/mmm MgB$_{2}$ theoretically encompasses contributions from both acoustic and optical modes. Notably, $\alpha ^{2} F$($\omega$) value is particularly pronounced in the optical phonon mode, resulting in a significant integration of $\lambda$, as illustrated in Fig. 7(a). We observed that the P6/mmm MgB$_{2}$ has a $\lambda$ value of 0.80. Now, let’s shift our focus to the stable structures, namely, the $R{\bar{3}}m$ Mg$_{0.333}$Mo$_{0.667}$B$_{2}$ and the *Immm* Mg$_{0.5}$Mo$_{0.5}$B$_{2}$ at ambient condition. Our findings indicate that $\alpha ^{2} F$($\omega$) encompasses contributions spanning from acoustic to optical modes, which are illustrated in  Figs. 7(b) and 7(c). Similarly, the $I{\bar{4}}m2$ Mg$_{0.5}$Mo$_{0.5}$B$_{2}$ structure also demonstrates $\alpha ^{2} F$($\omega$) contributions spanning the acoustic to optical modes at a pressure of 50 GPa, as depicted in Fig. 7(d). Subsequently, we theoretically derived the EPC constant, denoted as $\lambda$, by using the Eq. (3). This $\lambda$ can be determined by integrating $\alpha ^{2} F$($\omega$). Broadly speaking, the $\lambda$ solution exhibits a sharp increase in the low-frequency phonon mode, followed by a gradual increase in the medium-frequency phonon mode. It then experiences a slight rise up to the highest phonon frequency, thus showcasing the overall integrated $\lambda$. Consequently, the integrated $\lambda$ of $R{\bar{3}}m$ Mg$_{0.333}$Mo$_{0.667}$B$_{2}$, *Immm* Mg$_{0.5}$Mo$_{0.5}$B$_{2}$, and the I${\bar{4}}$m2 Mg$_{0.5}$Mo$_{0.5}$B$_{2}$ is 0.62, 0.32, and 0.36, respectively. As previously mentioned, the DOS significantly influences the $\lambda$. Notably, the DOS of $R{\bar{3}}m$ Mg$_{0.333}$Mo$_{0.667}$B$_{2}$ is predominantly concentrated at the Fermi level, which is directly reflected in the $\lambda$ value. This observation clarifies why the $\lambda$ is notably high in this case. Moreover, it is intriguing to note that the EPC constant’s magnitude is governed by the DOS. This raises the possibility that the Mo concentration plays a crucial role, given its contribution at the Fermi level. This factor may hold importance in understanding the superconducting mechanism.

3 $$\begin{aligned} \lambda (\omega ) = 2 \int _{0}^{\omega }d\omega '\frac{\alpha ^{2}F(\omega ')}{\omega '}, \end{aligned}$$

To assess the $T_{c}$, we employed the AD equation with $\mu ^{*}$= 0.10. Initially, we examined the cd $T_c$ of the P6/mmm MgB$_{2}$, and found it to exhibit superconductivity with a $T_c$ of 34 K. This result aligns well with experimental observations^1^. Subsequently, we determined the $T_c$ of the $R{\bar{3}}m$ Mg$_{0.333}$Mo$_{0.667}$B$_{2}$ and the *Immm* Mg$_{0.5}$Mo$_{0.5}$B$_{2}$. Our comprehensive analysis also indicates superconductivity in these compounds, with the $T_c$ values of 7.4 K and 0.43 K, respectively, at ambient pressure. Notably, our study reveals that the $T_c$ of the $R{\bar{3}}m$ Mg$_{0.333}$Mo$_{0.667}$B$_{2}$ and the *Immm* Mg$_{0.5}$Mo$_{0.5}$B$_{2}$ are relatively low compared to the $T_c$ of the P6/mmm MgB$_{2}$. The underlying reason for this discrepancy can be attributed to our critical analysis of the the EPC and $\omega _{log}$ values, primarily influenced by the presence of Mo atoms. This suggests that the presence of Mo may be one of the factors limiting the increase in $T_c$ In addition to $\mu ^{*}$= 0.10, these findings may hold relevance even when the $\mu ^{*}$= 0.13 is not universally applicable to all simple metals^53^, as it is primarily suited for transition metals. To some extent, however, it is reasonable to anticipate that adopting $\mu ^{*}$= 0.13 in theoretical predictions might result in an enhancement of *T*_*c*_. At this juncture, particular attention should be directed towards the $R{\bar{3}}m$ Mg$_{0.333}$Mo$_{0.667}$B$_{2}$ due to its substantially higher $T_{c}$ compared to the *Immm* Mg$_{0.5}$Mo$_{0.5}$B$_{2}$. Specifically, the $R{\bar{3}}m$ Mg$_{0.333}$Mo$_{0.667}$B$_{2}$ exhibits superconductivity with a $T_{c}$ of 5.34 K and it is noteworthy that its $T_c$ decreases when $\mu ^{*}$= 0.13 is employed. It is important to highlight that the $T_{c}$ values observed in Mg$_{0.333}$Mo$_{0.667}$B$_{2}$ and Mg$_{0.5}$Mo$_{0.5}$B$_{2}$ are significantly lower than that of MgB$_{2}$, where $\mu ^{*}$= 0.10 is used^10^. As indicated by previous theoretical findings^10^, the high DOS of boron at the Fermi level plays a pivotal role in achieving high-$T_{c}$ in MgB$_{2}$. In contrast, within our system, it appears that the DOS of boron in Mg$_{0.333}$Mo$_{0.667}$B$_{2}$ and Mg$_{0.5}$Mo$_{0.5}$B$_{2}$ may not be sufficiently high in comparison to MgB$_{2}$, thereby limiting their *T*_*c*_ potential. However, it becomes intriguing to further investigate the $T_{c}$ behavior of these compounds under compression. Beyond ambient pressure, up to 25 GPa, we have observed that the $R{\bar{3}}m$ Mg$_{0.333}$Mo$_{0.667}$B$_{2}$  exhibits superconductivity with a $T_{c}$ of 5.3 K, while, the $T_{c}$ of the $I{\bar{4}}m2$ Mg$_{0.5}$Mo$_{0.5}$B$_{2}$ reached 1.12 K at 25 K. Beyond 25 GPa, there is a general trend of decreasing $T_{c}$ with increasing pressure. A summary of the main results is presented in Table  2. Therefore, considering the impact of compression, it is reasonable to speculate that these structures have the potential for superconductivity up to at least 50 GPa. The primary effect of compression suggests that increasing pressure may not be conducive to maintaining the superconducting state in these structures, as there is a clear trend toward transitioning from a superconducting state to a normal metallic state under compression. As previously mentioned in experimental findings^23^, despite the discrepancy between experimental observations and our theoretical explanations, particularly regarding the evolution of $T_{c}$ at ambient conditions, one aspect related to the concentration of Mo is noteworthy. It is evident that the specific crystal structure plays a critical role in this context. This implies that the evolution of $T_{c}$ may indeed be influenced by the concentration of Mo, especially when the Mo atom’s placement aligns with the lattice site. Consequently, this could explain why a reduced $T_{c}$ was observed in experimental studies. While this analysis provides a straightforward starting point for understanding the relationship, it also poses a challenge to the assumptions made. Therefore, we propose that the influence of Mo concentration on MgB$_{2}$ warrants further experimental investigations.

Regarding the bonding environment of the R${\bar{3}}$m Mg$_{0.333}$Mo$_{0.667}$B$_{2}$, the *Immm* Mg$_{0.5}$Mo$_{0.5}$B$_{2}$, and the $I{\bar{4}}m2$ Mg$_{0.5}$Mo$_{0.5}$B$_{2}$, we have explored their electronic properties using the electron localization function (ELF) method.^54^. The ELF provides insights into the distribution of electrons within crystals relative to a uniform electron gas of the same density, with ELF values ranging between 0 and 1, indicating the tendency of electron pairing. ELF values are always positive. To begin, let’s examine the bonding environment of $R{\bar{3}}m$ Mg$_{0.333}$Mo$_{0.667}$B$_{2}$. We illustrate this environment in the (110) atomic plane, where the ELF reveals that electrons tend to accumulate more favorably between Mo and B atoms than between Mg and B atoms, as depicted in Fig. 8(a). In the Immm Mg$_{0.5}$Mo$_{0.5}$B$_{2}$, the ELF analysis reveals that electrons tend to accumulate more favorably between Mo and B atoms than between Mg and B atoms in both the (011) and (100) atomic planes, as depicted in Fig  8(b). Similarly, in the $I{\bar{4}}m2$ Mg$_{0.5}$Mo$_{0.5}$B$_{2}$, the ELF analysis in the (100) atomic plane indicates that electrons preferentially accumulate between Mo and B atoms, while they do not favorably accumulate between Mg and B atoms, as shown in the Fig  8(c). Furthermore, a strong covalent bond is indicated by the significant electron accumulation between B and B atoms. It is noteworthy that the bonding environment of Mo-B, Mg-B, and B-B exhibits characteristics of both ionic and covalent bonds^17,55–60^. Considering the bonding environment’s influence on the system, the electron contributions between Mo and B atoms appear to be significantly connected with the observed $T_{c}$. In our research, we propose that the emergence of superconductivity could potentially be observed in new metallic phases present in the Mg_1−x_Mo_x_B_2_ system, under both ambient and high-pressure conditions. If this hypothesis holds true, it would be worthwhile to explore further through analyses using energy dispersive X-ray spectroscopy (EDS) and X-ray photoelectron spectroscopy (XPS) to gain insights into the underlying chemical bonding^61^.

## Conclusion

In summary, we present the discovery of novel metallic phases within the Mg$_{1-x}$Mo$_{x}$B$_{2}$ system, utilizing a combination of first-principles cluster expansion and particle swarm optimization techniques. Among the structures located along the convex hull, Mg$_{0.667}$Mo$_{0.333}$B$_{2}$, Mg$_{0.5}$Mo$_{0.5}$B$_{2}$, and Mg$_{0.333}$Mo$_{0.667}$B$_{2}$, exhibit the lowest enthalpy, and they remain stable at ambient pressure. Remarkably, these structures maintain their structural stability even under high pressures, extending up to at least 50 GPa. An analysis of the density of states reveals that the electronic states of Mg$_{0.333}$Mo$_{0.667}$B$_{2}$ near the Fermi level exhibit a high sensitivity to electron-phonon coupling when compared to Mg$_{0.5}$Mo$_{0.5}$B$_{2}$. Consequently, Mg$_{0.333}$Mo$_{0.667}$B$_{2}$ demonstrates a considerably high *T*_*c*_ of 7.4 K, even at ambient pressure. These findings highlight the significance of molybdenum substitution in magnesium diboride as it pertains to achieving high $T_{c}$ superconductors. We anticipate that our results will encourage further experimental investigations in this category of superconducting materials.

## Data Availability

The data that support the findings of this study are available from the corresponding author upon reasonable request.
